# Title: Identification of Redox State Based on the Difference in Solvation Dynamics

**DOI:** 10.1002/open.202400278

**Published:** 2025-03-18

**Authors:** Yasuhiro Kato, Jelena Muncan, Yoshinori Hirano, Hiroko Yamamoto, Roumiana Tsenkova, Masato Yasui

**Affiliations:** ^1^ School of Medicine Keio University 35 Shinanomachi, Shijyuku-ku Tokyo 160-8582 Japan; ^2^ Keio Global Research Institute Keio University 2-15-45 Mita, Minatou-ku Tokyo 108-8345 Japan; ^3^ Graduate School of Agricultural Science Kobe University 1-1 Rokkodai, Nada-ku Kobe 657-8501 Japan; ^4^ Faculty of Science and Technology Keio University 3-14-1 Hiyoshi, Kohoku-ku Yokohama 223-8522 Japan.

**Keywords:** Redox, near infrared spectroscopy, water structure, biological processes, glutathione, nicotinamide adenine dinucleotide

## Abstract

Oxidation‐reduction (Redox) reactions are crucial for many biological processes, yet there is no method available to evaluate redox states in a non‐invasive, continuous manner. Here we introduce a novel approach to distinguish between reduced and oxidized states of glutathione (GSH and GSSG, respectively) using aquaphotomics near‐infrared (NIR) spectroscopy and multivariate analysis. We identified clear differences in NIR spectra reflecting not only glutathione itself, but different redox states of glutathione based on the spectral features of water molecular conformations interacting with the reaction site. Molecular dynamic simulations also revealed the difference in water molecule coordination and hydration numbers around the reaction site. This approach not only sheds light on the significance of water molecules in redox reactions but also enables non‐destructive, continuous assessment of redox states, with potential applications for bioreactor optimization.

## Graphical abstract

Redox reactions play a crucial role in biological systems, yet non‐invasive, continuous evaluation remains challenging. This study utilizes aquaphotomics NIR spectroscopy and multivariate analysis to differentiate reduced and oxidized glutathione (GSH/GSSG) by analyzing water molecular conformations depicted as spectral pattern. Key spectral features at 1362 nm and 1381 nm highlight redox state differences, supported by molecular dynamics simulations. This method provides a novel, non‐destructive tool for real‐time redox monitoring with potential applications in bioreactors.

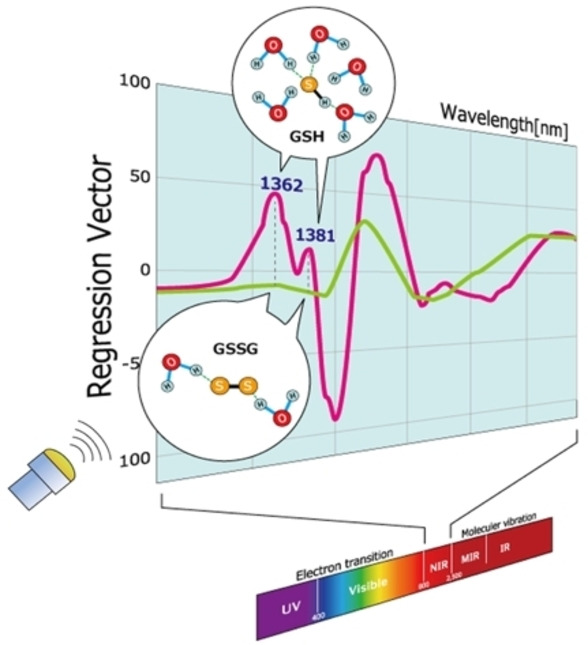

## 1 Introduction

Oxidation‐reduction (Redox) reactions are critical in determining the status of biological systems as well as in the optimization of bioreactor performance.^[1]^ Imbalances in redox homeostasis may lead to damage of critically important biomolecules like DNA, proteins, and lipids, and may contribute to onset of diseases such as cancer, neurodegenerative disorders, and cardiovascular diseases.[^2^, ^3^, ^4^] Glutathione, a crucial antioxidant tripeptide, exists in both reduced (GSH) and oxidized (GSSG) forms, whose ratio serves as a measure of cellular oxidative stress.^[1]^ The concentration of GSH has been inversely correlated with the severity of neurological disorders and cognitive impairment, showing its essential role in maintaining cellular health.^[4]^ Traditional methods for evaluating redox reactions often involve invasive procedures and complex sample preparation.^[1]^ Although fluorescence dyes have been developed for detecting both GSH and GSSG, their utilization requires specialized equipment, limiting accessibility. In contrast, non‐destructive spectral analyses such as Raman and infrared spectroscopy offer distinct advantages. Among these techniques, near‐infrared (NIR) spectroscopy provides valuable insights into molecular structures and molecular interactions, and its particular advantage is that is allows non‐destructive, non‐invasive measurements with little or no sample preparation.^[5]^ The systematic approach referred to as “Aquaphotomics” has been developed by focusing on dynamic structures of water molecular organization.^[6]^ This approach can provide the important information about dynamic interaction between solutes and water molecules in solution. Therefore, we employed aquaphotomics near‐infrared (NIR) spectroscopy to assess redox states that may reflect the interaction between water molecules and GSH or GSSG.

## 2 Results

### 2.1 Evaluating Oxidation and Reduction Reactions Involving Glutathione Using Near‐Infrared Spectroscopy.

**Figure 1:**
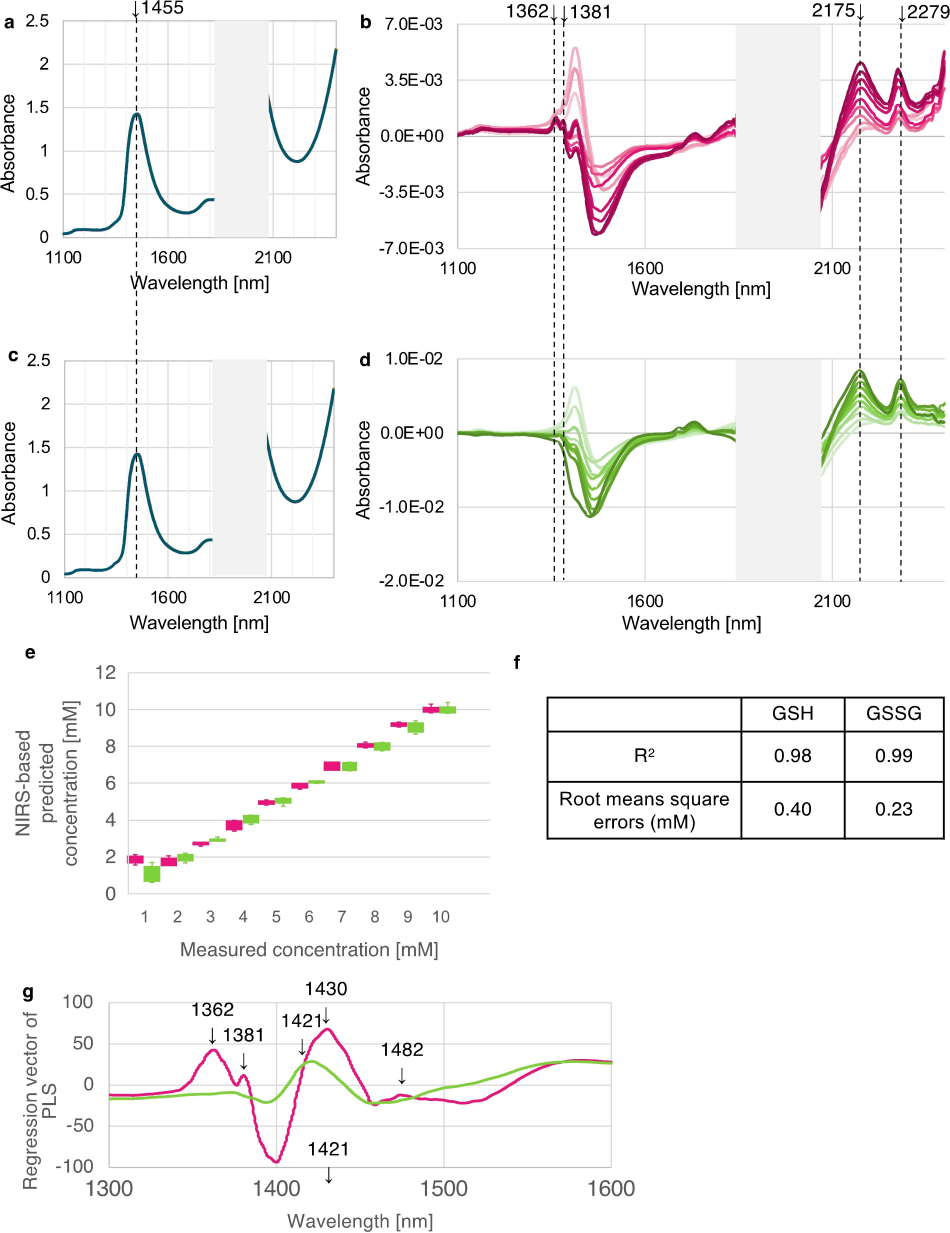
**The NIR spectra show clear differences between GSH and GSSG through the application of difference spectra and PLSR analysis**. Raw spectra from 1–10 mM GSH (a) and (c) GSSG between 1100–2500 nm (excluding 1850–2050 nm). Spectra obtained by subtracting the average spectrum of PBS solution from the spectra of 1–10 mM glutathione solutions GSH (b, red line) and GSSG (d, green line), corresponding to concentration range. Data processed similarly to Figure 1a–1d, using raw spectra preprocessed (standardization, smoothing), outliers removed based on the values of Mahalanobis distance in PCA and PLSR to predict GSH or GSSG concentration. Model developed using OSC as a pre‐processing technique (1 component), validation was internal (leave‐one‐out cross‐validation). e) Y fit plot shows excellent agreement between NIRS‐predicted concentrations and actual concentrations. e) Prediction accuracy decreased at 1 mM, but was significantly smaller for 3 mM and 6 mM GSH/GSSG compared to other concentrations. f) NIRS accuracy was high, with coefficient of determination of 0.98‐0.99, close to 1. Root means square errors (RMSE) 0.40 mM (GSH) and 0.23 mM (GSSG), indicated good estimation accuracy. g) The NIRS regression vector on PLS of specific bands identifies reduced (GSH) and oxidized (GSSG) glutathione. The regression equation of GSSG/GSH shows glutathione characteristics in 1300–1600 nm region, the first overtone of water.

First, we investigated whether oxidation and reduction reactions can be evaluated using non‐staining NIR spectroscopy combined with multivariate analysis. We used glutathione solutions: GSH and GSSG in the 1–10 mM range. The raw data (Figure 1a and 1c), showed no discernible differences in discrimination or concentration between GSH and GSSG. However, after calculating the difference spectra by subtracting the NIR spectra of the PBS (phosphate‐buffered saline) background from the spectra of the sample solutions, a clear differentiation between GSH and GSSG emerged. In the 1300–1600 nm wavelength range, GSH‐specific peaks were evident, particularly around 1362 and 1381 nm (Figure 1b). These peaks were absent for GSSG (Figure 1d). In particular, the two peaks located at 1362 nm and 1381 nm indicate the presence of water of hydration in the first layer, termed the water solvation shell as supported by previous findings that link changes in NIR spectral features to variations in water structure in solvation shells.^[7]^ These results suggest that the distinction between GSH and GSSG forms might originate from differences in water of hydration surrounding ‐SH and S−S groups, respectively.

The difference in the negative peak intensity at ~1450 nm between GSH and GSSG is attributed to the distinct solvation shell structures.^[8]^ The disulfide bond formation in GSSG alters the hydrogen bonding network, resulting in a more pronounced negative peak compared to GSH.

On the other hand, in the wavelength range of 2200–2400 nm, the following two peaks are observed in both GSH and GSSG solutions: 2175 nm and 2279 nm. The peak at 2175 nm is assigned to a combination of NH‐stretching vibration and CONH2 groups associated with amide II, as well as combinations of amide I. ^[9]^ The peak at 2279 nm represents CONH_2_ groups and O−H, C−O stretching combinations.^[10]^ These spectral bands are sensitive to concentrations ranging from 1–10 mM (Figure 1b and 1d), supporting the validity of the method.

### 2.2 Quantitative Evaluation of GSH and GSSG Concentrations Using Near‐Infrared Spectroscopy.

Having successfully identified the difference between GSH and GSSG in the 1300–1600 nm range, we questioned whether it is possible to do quantitative analysis and determine their respective exact concentrations. Our model successfully predicted concentrations of GSH and GSSG in the first overtone of water region, 1300–1600 nm.^[5]^ Spectral data for the predictive model were processed in a manner analogous to that shown in Figure S1a–S1 h. This involved preprocessing of raw spectra (standardization, smoothing), exclusion of outliers based on the Mahalanobis distance in Principal Component Analysis (PCA), and application of Partial Least Squares Regression (PLSR) to the subtracted spectra to predict concentrations of GSH and GSSG.[^10^, ^11^] Subsequently, Y fit plot, showing the agreement between the actual and predicted values showed excellent correlation, except in the case of 1 mM GSH (Figure 1e). Predictive accuracy of the developed PLSR model was high, with determination coefficients ranging from 0.98 to 0.99. Root Mean Square Error (RMSE) values were 0.40 mM for GSH and 0.23 mM for GSSG (Figure 1f). The comparison of regression vectors of developed PLSR models for prediction of concentration of GSH and GSSG, identified wavelengths at 1362 nm and 1381 nm to distinguish between the two (Figure 1g), consistent with the difference spectra role in discriminating GSH and GSSG at 1300–1600 nm range (Figure 1g).

On the other hand, we focused on NIR‐based quantification of GSH and GSSG in mixed solutions. GSSG has twice the molecular weight of GSH, and therefore, mixed solutions of GSH and GSSG were prepared at half the molar concentration of GSSG relative to GSH. The mixed sample of GSH and GSSG was analyzed using Principal Component Regression (PCR) to predict GSH and GSSG concentrations.^[12]^ The Y‐fit exhibited a strong correlation between the predicted NIR concentration and the actual concentrations of GSH and GSSG (Figure S2a, b). The predictive accuracy of NIR spectroscopy was high, with a determination coefficient of 0.82 and RMSE values of 0.81 mM for GSH and 0.40 mM for GSSG, indicating a precise and reliable model (Figure S2c).

Regression analysis further highlighted critical wavelengths at 1362 nm and 1381 nm for distinguishing GSH and GSSG (Figure S2d, where the red line represents GSH and the green line represents GSSG). Based on these results, we can conclude that absorbance values at 1362 nm and 1381 nm are indicative of solvation dynamics and serve as key indicators for discriminating GSH and GSSG in mixed samples.

These findings demonstrate the feasibility of using NIR spectroscopy for assessing redox states in complex solutions.

### 2.3 Comparative Analysis of Local Water Molecular Dynamics in Redox Reactions by Molecular Dynamic Simulations.

**Figure 2:**
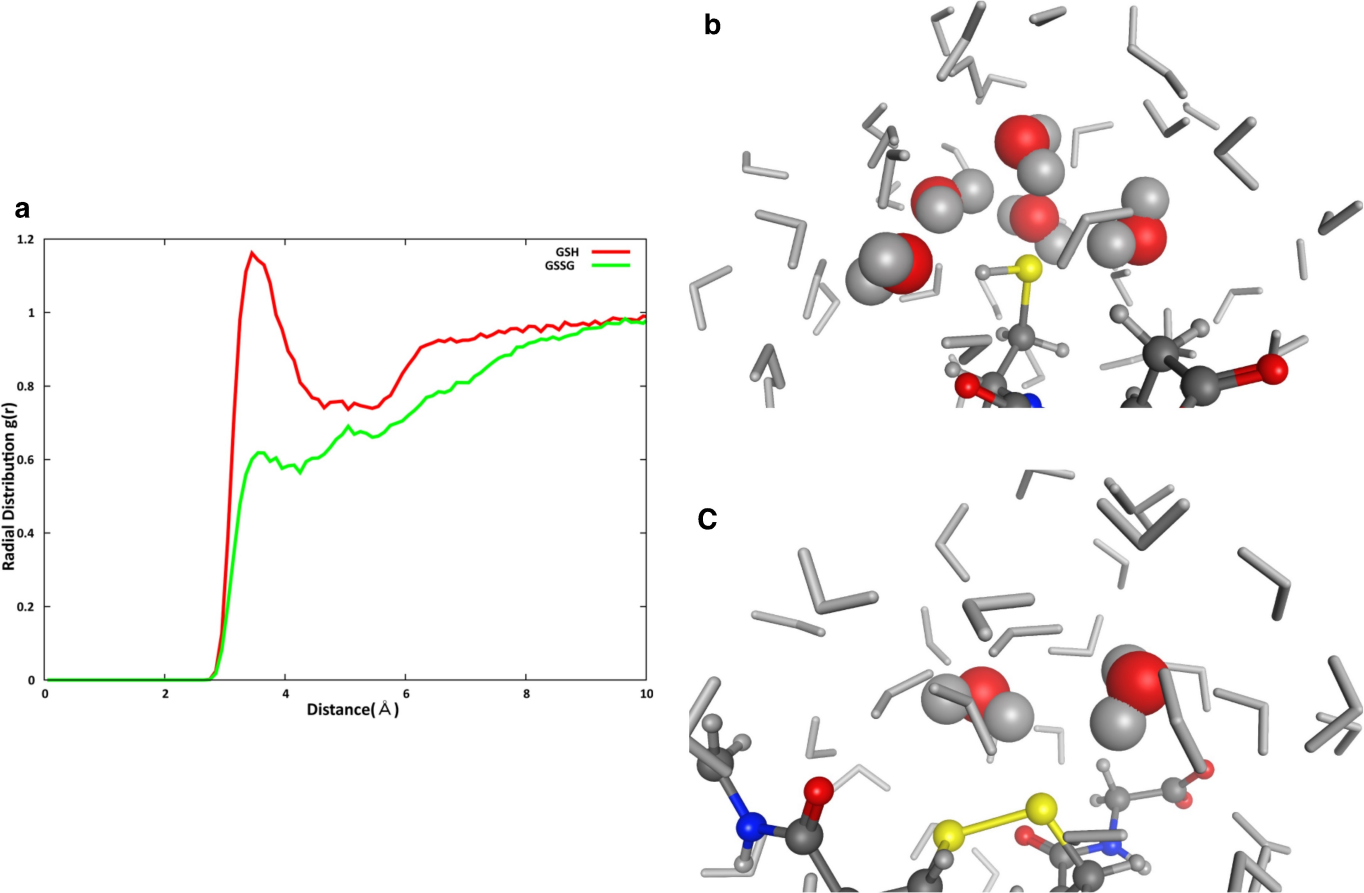
**Distribution of water molecules near sulfur in GSH and GSSG**. (a) Red/green lines show radial distribution functions (RDFs) of water oxygen (O) atoms from sulfur (S) atoms in GSH/GSSG, respectively. (b, c) Snapshots of GSH/GSSG models around sulfur atoms. Yellow, red, blue, gray, and white circles represent sulfur, oxygen, nitrogen, carbon, and hydrogen atoms, respectively. Water molecules close or far from the sulfur atom shown in space‐filling (red/gray) and stick models (gray).

The near‐infrared spectroscopic data suggest differences in the distribution and coordination number of surrounding water molecules on the sulfur atoms of GSH and GSSG. To further investigate water coordination, we conducted molecular dynamic (MD) simulations and compared interactions between the sulfur atom in each glutathione molecule and adjacent water molecules.^[13]^ The radial distribution function (RDF) revealed a considerable difference in the distribution of water molecules around the sulfur atoms of GSH and GSSG (Figure 2a).^[14]^ The first peak, situated approximately 3.8 Å from the sulfur atom, was approximately twice as high in GSH compared to GSSG, indicating a difference in the number of water molecules interacting with the sulfur atom. A second peak was observed at 6.0 Å (Figure 2a). In contrast, the distribution of water molecules located more than 10.0 Å from the sulfur atom in both GSH and GSSG was nearly identical and resembled bulk water molecules (Figure 2a).

To understand the distributions of water molecules around a sulfur atom in GSSG and GSH and their behavior, we analyzed hydrogen bonds between a sulfur atom and water molecules by defining the interaction score. This score is the sum of hydrogen bonds between a sulfur atom in GSH or GSSG and water molecules, weighted by their residence time during the last 100 ns (10,000 frames).

The interaction scores for the oxygen donor in water molecules and the sulfur acceptor (OH−S) in GSH and GSSG were 6,133 and 5,977, respectively. Similarly, the interaction scores for the sulfur donor and the oxygen acceptor in water molecules (SH−O) in GSH and GSSG were 5,540 and 0, respectively.

We also calculated the interaction scores of GSSG and two GSH molecules to equalize the number of sulfur atoms. The total interaction scores for OH−S in GSSG and two GSH molecules were 11,811 and 12,266, respectively, while for SH−O, they were 0 and 10,454, respectively. These results suggest that when normalized per sulfur atom, GSH exhibits approximately twice the total interaction score compared to GSSG.

This phenomenon can be attributed to the sulfur atom in the GSH model, which serves as both a donor and an acceptor for hydrogen ions, whereas the sulfur atom in GSSG primarily functions as a hydrogen bond acceptor (Figure 2b and 2c). Due to the dual roles of GSH and the presence of water molecules around the sulfur atom, the SH group can undergo transformation into an S‐group simultaneously with the release of a proton, thereby acting as a reducing agent. The arrangement of water molecules in this region in GSH displayed slightly greater order than that in GSSG (Figure 2b and 2c).

Our findings suggest that the sulfur atom in GSH has a greater tendency to act as a proton donor compared to that in GSSG, which may explain observed differences in NIR spectra of GSH, and GSSG, and mixed solutions.

### 2.4 NIR Spectroscopy to Discern NADH and NAD^+^ Redox States: Insights from Hydration Structures and Critical Wavelengths.

**Figure 3:**
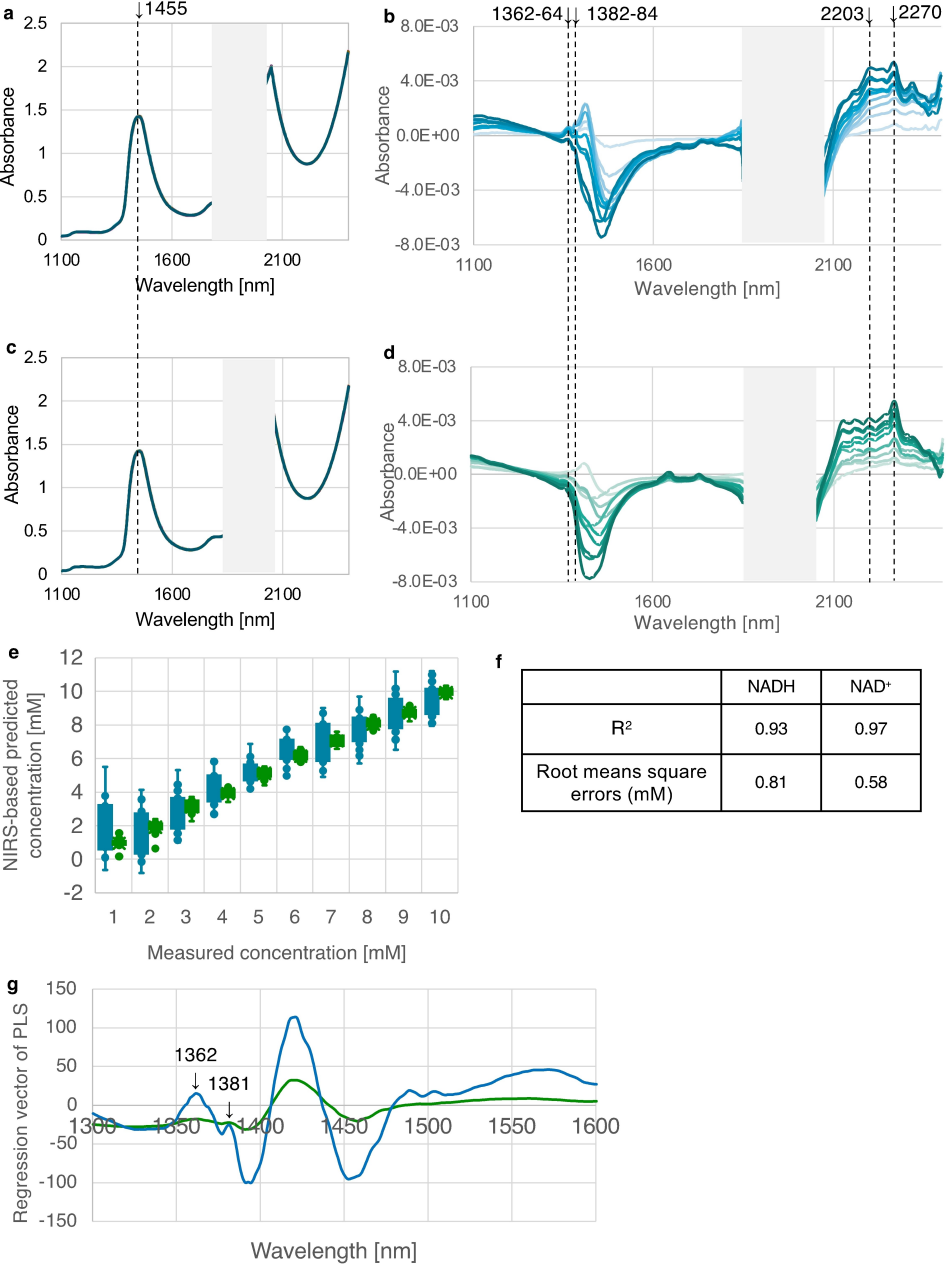
**The common spectrum of redox potential for the reduced (NADH) or oxidized form (NAD^+^) employs subtracted spectra and Partial Least Squares (PLS) regression analysis of the region from 1300 ‐ 1600 nm**. Raw spectra are from 1–10 mM NADH (a) or NAD^+^ (c) between 1100–2500 nm without 1850–2050 nm. The spectrum obtained by subtracting PBS from 1–10 mM NADH (b, blue line) or NAD^+□^(d, green line) is represented by color. Data for the prediction model were processed using raw spectra preprocessed (standardization, smoothing), outliers removed using Mahalanobis distance on the Principal Component Analysis (PCA) and applying Partial Least Squares Regression (PLSR) to subtracted spectra to predict NADH or NAD^+^ concentrations. The prediction model was validated using OSC (1 component, leave‐one‐out cross‐validation). e) As a result, the Y fitting showed that the predicted concentration by NIRS correlated with the actual concentration, excluding 1 mM NADH. (F) Prediction accuracy of NIRS was high, with a determination coefficient of 0.93‐0.97. (**F**) The root mean square error (RMSE) was 0.81 mM (NADH) and 0.58 mM (NAD^+^), suggesting a model with good estimation accuracy. g) The NIRS regression vector is a display on the Partial Least Squares (PLS) of specific bands to identify reduced (NADH) and oxidized (NAD^+^) forms. From the regression equation of NADH/NAD^+^, the characteristics of glutathione (GSH or GSSG) are shown in the 1300–1600 nm region, which is the first overtone of water (various vibration bands of O−H bond).

To examine whether our findings are specific to GSH/GSSG or serve as general indicators of oxidation‐reduction reactions, we explored hydration structures of NADH (nicotinamide adenine dinucleotide, reduced form) and NAD^+^ (nicotinamide adenine dinucleotide, oxidized form) in biological redox reactions.^[15]^ Raw spectra did not exhibit clear distinctions between NADH and NAD^+^ (Figure 3a and 3c). However, subtracting the spectra showed a clear difference between NADH and NAD^+^. In the 1300–1600 nm wavelength range, NADH‐specific peaks were evident, particularly around 1362–64 nm (Figure 3b). These peaks were absent for NAD^+^ around 1382–84 nm (Figure. 3D). The Y fitting exhibited a correlation between the predicted NIR concentration and the actual concentration (Figure 3e). Predictive accuracy of NIR spectroscopy was high, with determination coefficients ranging from 0.93 to 0.97 and RMSE values of 0.81 mM for NADH and 0.58 mM for NAD^+^, indicating precise and reliable models (Figure 3f). Regression analysis also highlighted critical wavelengths at 1362 and 1381 nm for discriminating between NADH and NAD^+^ (Figure 3g). Based on these results, we concluded that absorbance values at 1362 nm and 1381 nm are indicative of solvation dynamics rather than of substances themselves and can be used as common indicators for redox states.

## 3 Conclusions

In this study, we employed NIR spectroscopy combined with multivariate analysis for non‐invasive assessment of redox states. Comparison of GSH and GSSG solutions revealed differences in hydration structures, particularly at 1362 nm and 1381 nm, implying unique environments around SH and S−S sites. Molecular dynamic simulations also revealed differences in water molecule coordination and hydration numbers around the reaction site. Similar hydration structures in GSH/GSSG and NADH/NAD^+^ suggest their roles in proton transfer and redox activities. Based on these results, wavelengths at 1362 nm and 1381 nm, indicative of differences in hydration rather than substances themselves, may be used as common indicators for redox states. We therefore propose that differences in local hydration can serve as useful indicators of oxidation‐reduction state.

Traditional methods such as NMR, HPLC, or colorimetric assays, while effective in substance identification, often require preprocessing and expensive equipment.^[14]^ In contrast, NIR‐based approaches are non‐destructive and non‐staining, offering convenient implementation. Furthermore, our method enables stable measurement of samples using NIR spectra without any particular, laborious sample preparation, reducing errors related to sample preparation variations often observed in biological samples.[^17^, ^18^] Integration of multivariate analysis and Molecular Dynamics illustrates the ability to depict numbers and arrangements of water molecules involved in hydration (Figure 2b, c and Figure S3a, b)). The structure of water molecules around the substances, rather than substances themselves, enables evaluation of redox states.

GSH, unlike GSSG, has a H atom covalently bonded to the S atom, likely forming additional hydrogen bonds with O atoms of solvent water molecules. This local variation in hydration patterns, evident in NIR spectra, suggests a solvation shell surrounding GSH′s sulfur‐hydrogen region, leading to stronger hydration structure formation than in GSSG, as seen in the peaks at 1362 and 1381 nm.[^6^, ^7^, ^19^, ^20^]

Note that the regions at 1362 nm and 1381 nm signify the water solvation shell. The 1362‐nm region is associated with hydration in the first layer, where water molecules act as proton receptors during reduction^[21]^ and the 1381‐nm region represents hydration from the first to third layers, or may indicate the presence of hydronium ion (H_3_O)^+^.^[22]^ While such hydration structures are common around solutes, the exclusive presence of peaks in GSH at these wavelengths (Figure 1g), in contrast to GSSG (Figure 1g), suggests a unique hydration structure exclusive to GSH.

Molecular snapshots support the presence of four to five water molecules in the first solvation layer of sulfur atoms (Figure 2b, c and Figure S3a, b). Additionally, the greater involvement of water molecules in GSH‐related hydration processes, compared to GSSG, is implied by the height of the 3.8 Å peak in RDF and broader bandwidths at 1362 and 1381 nm in NIR (Figure 2a). Proton transfer in redox reactions relies on local water molecules and the overall hydration environment. These peaks indicate that the (H_3_O)^+^ serves as a proton source, modulating electron transfer between reactants.[^19^, ^22^] Exploring properties at 1362 and 1381 nm for anti‐oxidative activity bands associated with proton hydrates, the future work will focus on verifying proton transfer by quantum chemical calculations.

## Experimental Section/Methods

**Sample Preparation**: Glutathione samples were prepared from pure glutathione at concentrations ranging from 1 to in 10 mM increments for both GSH (Fujifilm: 077–02011) and GSSG (Fujifilm: 079–03333) inphosphate‐buffered saline (PBS). Mixed solutions were prepared as follows: 1 mM GSH: 4.5 mM GSSG, 2 mM GSH: 4 mM GSSG, 3 mM GSH: 3.5 mM GSSG, up to 9 mM GSH: 0.5 mM GSSG. Because GSSG has twice the molecular weight of GSH, the mixed solutions were prepared with GSSG at half the molar concentration of GSH. The pH was adjusted to 7.5 using 5 M NaOH. ß‐NADH and ß‐NAD^+^ samples were diluted to concentrations ranging from 1 to 10 mM, in 1 mM increments in PBS (ß‐NADH: Fujifilm: 301–50453, ß‐ NAD^+^: Fujifilm: 304–50443).

**NIRS measurements**: NIR transmittance spectra were acquired using Fourier Transform NIR (FT‐NIR) spectrometer (MPA Bruker Optics, Tokyo, Japan)) with a sample cell having a 1‐mm path length. Spectra ranged from 1100 nm to 2400 nm, Measurements were taken for glutathione samples (GSH, GSSG, ß‐NADH, and ß‐NAD^+^) at 1–10 mM concentrations. For each sample, 800 scans (32 scans × 25 repeat sample measurements) were taken.

**Data Analysis**: Multivariate analysis was performed using Pirouette ver. 4.5 (Infometrix, Inc. WA, USA) and MATLAB (Version 7.1; The MathWorks, MA, USA). Spectra were processed between 1100–1850 nm and 2050–2400 nm, excluding regions below 1100 nm and 1850–2050 nm, due to noise and high absorbance issues. Baseline correction was performed using Standard Normal Variate (SNV) transformation, noise reduction was achieved using Savitzky‐Golay 2nd degree polynomial filter, and spectrum similarity was assessed using Mahalanobis distance in Principal Component Analysis (PCA).^[6]^ Difference spectra alongside PCA were used for outlier detection and removal, while Partial Least Squares Regression (PLSR) was employed to evaluate spectral variations and to correlate them with GSH and GSSG concentrations.[^6^, ^11^] These preprocessing techniques were primarily based on the Pirouette ver. 4.5 (Infometrix, Inc., WA, USA) algorithms. Principal Component Regression (PCR) was used for concentration estimation and classification modeling in mixed solutions, specifically within the 1300–1600 nm wavelength range without preprocessing. Furthermore, a prediction model was constructed, and validation was performed using leave‐five‐spectra‐out cross‐validation.^[8]^

**Molecular Dynamics Simulations**: Model systems for molecular dynamic (MD) simulations were constructed using GSH and GSSG. A TIP3P water box of 68x68x68 Å^3^ was generated around GSH and GSSG molecules. System sizes of these models were about 26,000 atoms each. The general amber force field (GAFF) was used for GSH and GSSG.^[23]^ Partial charges for GSH and GSSG were calculated at the HF/6‐31G (d) level using Gaussian 09 software (Gaussian Inc.) and the restrained electrostatic potential method.^[24]^ The periodic boundary condition was applied to the initial system, and temperature and pressure were kept constant using the Nosè–Hoover thermostat.^[24]^ and the Parrinello–Rahman barostat,^[27]^ respectively. The linear constraint solver (LINCS) algorithm^[27]^ was applied to the covalent bonds, with an integration time step of 2.0 fs taken into consideration. Long‐range Coulomb interactions were treated using the particle mesh Ewald method^[28]^ and the direct space cutoff distance was set to 10.0 Å. Van der Waals interactions were calculated using a switched cutoff between 8.0 and 10.0 Å. The NPT ensemble (P=1 bar and T=298 K) was used for MD simulations. Whole MD simulations were performed using GROMACS version 5.0.6 software.[^28^, ^29^, ^30^, ^31^, ^32^, ^33^]

## Conflict of Interests

The authors declare no conflict of interest.

4

## Supporting information

As a service to our authors and readers, this journal provides supporting information supplied by the authors. Such materials are peer reviewed and may be re‐organized for online delivery, but are not copy‐edited or typeset. Technical support issues arising from supporting information (other than missing files) should be addressed to the authors.

Supporting Information

## Data Availability

The data that support the findings of this study are available on request from the corresponding author. The data are not publicly available due to privacy or ethical restrictions.
