# Supplementary material for: Title: Identification of Redox State Based on the Difference in Solvation Dynamics
Source: ChemistryOpen. 2025 Mar 18;14(5):e202400278. doi: 10.1002/open.202400278 (PMC12075091; doi:10.1002/open.202400278)
Supplement: Supplementary file 1 — Supporting Information [file OPEN-14-e202400278-s001.pdf]

ChemistryOpen

Supporting Information

Title: Identification of Redox State Based on the Difference in Solvation Dynamics

Yasuhiro Kato,* Jelena Muncan, Yoshinori Hirano, Hiroko Yamamoto, Roumiana Tsenkova, and Masato Yasui*

Supporting Information

Identification of Redox State Based on the Difference in Solvation Dynamics

Yasuhiro Kato[1,2], Jelena Muncan[3] Yoshinori Hirano[4], Hiroko Yamamoto [4], Roumiana Tsenkova[3], Masato Yasui[1,] **

Supplementary Text

Materials

Glutathione samples were prepared from pure glutathione at concentrations ranging from 1 to in 10 mM increments for both GSH (Fujifilm: 077-02011) and GSSG (Fujifilm: 079-03333) in phosphate-buffered saline (PBS). Mixed solutions were prepared as follows: 1 mM GSH: 4.5 mM GSSG, 2 mM GSH: 4 mM GSSG, 3 mM GSH: 3.5 mM GSSG, up to 9 mM GSH: 0.5mM GSSG. Because GSSG has twice the molecular weight of GSH, the mixed solutions were prepared with GSSG at half the molar concentration of GSH. The pH was adjusted to 7.5 using 5 M NaOH.

Methods

Near Infrared (NIR) spectroscopy: NIR transmittance spectra were acquired using FT-NIR spectrometer (MPA Bruker Optics, Tokyo, Japan) with a sample cell having 1-mm path length. Spectra were acquired in the range from 1100 nm to 2400 nm, excluding 1850-2050 nm. Measurements were acquired for glutathione samples (GSH, GSSG) at 1-10 mM and cellular lysates adjusted to 1 mg/mL. Each sample was scanned 800 times (32 scans x 25 repeat sample measurements).

Data Analysis: Multivariate analysis was performed using Pirouette ver. 4.5 (Infometrix, WA, USA) and MATLAB (Version 7.1; The MathWorks, MA, USA). Spectra intervals were processed between 1100–1850 nm and 2050-2400 nm, excluding regions below 1100 nm and 1850-2050 nm due to noise and high absorbance issues. Baseline correction was performed using Standard Normal Variate (SNV) transformation, the noise reduction by using Savitzky-Golay 2nd degree polynomial filter, and spectrum similarity was assessed using Mahalanobis distance in Principal Component Analysis (PCA).[6] Difference spectra and PCA were used for outlier detection and removal, while Partial Least Squares Regression (PLSR) was employed to evaluate spectral variations and to correlate them with GSH and GSSG concentrations.[6,11] These preprocessing techniques were primarily performed using the Pirouette ver. 4.5 (Infometrix, Inc., WA, USA) algorithms. Principal Component Regression (PCR) was used for concentration estimation and classification modeling in mixed solutions, specifically within the 1300–1600 nm wavelength range without preprocessing. Furthermore, a prediction model was constructed using the "Validation was performed using leave-five-spectra-out cross-validation.[8]

Result and discussion

Consideration of outlier removal and preprocessing for optimization of near-infrared spectroscopy (NIRS) spectra. To perform analysis of NIRS spectra, preprocessing is required to emphasize concentration-dependent changes while removing noise and to correct the

baseline . In this study, data obtained from 25 measurements for each sample (different concentrations) were used. When considering the entire spectra, all exhibit similar profiles (Figure 1a and 1c). However, when divided into concentration groups, principal component analysis of 25 spectra of GSH (1mM) showed that spectra grouped together for each repeated measurement (Figure S1a). In particular, spectra from the first measurement tended to be shifted to the right compared to subsequent spectra. There were no significant differences observed between the 3rd and 5th measurements, which tended to shift toward the left. Therefore, Mahalanobis distance for the 25 spectra is shown in Figure S1b. As a result, it was confirmed that the Mahalanobis distance varies depending on the number of repeated measurements. Since the threshold was set at 2.45 from the chi-square distribution, it is easier to remove spectra from the first measurement as outliers, and further adjustments beyond those already indicated are made for the remaining concentrations of GSH and GSSG. Results are summarized in Figure S1c and S1d.
After outlier removal, preprocessing included transformation using SNV and noise removal using a Savitzky-Golay filter, and subtraction of solvent information by the difference spectrum method.[6] As a result, spectral profiles of GSH and GSSG obtained using only the difference spectrum method are shown in Figure S1e and S1f. Subsequently, spectral profiles of GSH and GSSG after SNV-transformation, smoothing, and solvent subtraction are shown in Figure S1g and S1h. Baseline correction by SNV-transformation and reduction of high-frequency noise by smoothing emphasized the peaks that were previously not easy to detect. In particular, significant corrections to the baseline were made around 1300-1380 nm for GSH, and peaks around 1360 and 1380 nm that were previously hidden, became prominent. Although high-frequency noise was observed in both GSH and GSSG, the effect of smoothing with the Savitzky-Golay filter was particularly pronounced for GSH. Since GSSG already exhibited concentration-dependent changes in the raw data, effects were less pronounced compared to GSH, but peaks around 1400-1430 nm were emphasized, and high-frequency noise around 1450 nm and in other regions was reduced, confirming the effectiveness of preprocessing.

NIR-Based Quantification of GSH and GSSG in Mixed Solutions Using Principal Component Regression (PCR). GSSG has twice the molecular weight of GSH, and therefore, mixed solutions of GSH and GSSG were prepared at half the molar concentration of GSSG relative to GSH. The mixed sample of GSH and GSSG was analyzed using Principal Component Regression (PCR) to predict GSH and GSSG concentrations.[8] The Y-fit exhibited a strong correlation between the predicted NIR concentration and the actual concentrations of GSH and GSSG (Figure S2a, b). The predictive accuracy of NIR spectroscopy was high, with a determination coefficient of 0.82 and RMSE values of 0.81 mM for GSH and 0.40 mM for GSSG, indicating a precise and reliable model (Figure S2c).
Regression analysis further highlighted critical wavelengths at 1362 nm and 1381 nm for distinguishing GSH and GSSG (Figure S2d, where the red line represents GSH and the green line represents GSSG). Based on these results, we conclude that absorbance values at 1362 nm and 1381 nm are indicative of solvation dynamics and serve as key indicators for discriminating GSH and GSSG in mixed samples. These findings demonstrate the feasibility of using NIR spectroscopy for assessing redox states in complex solutions.

Figure S1

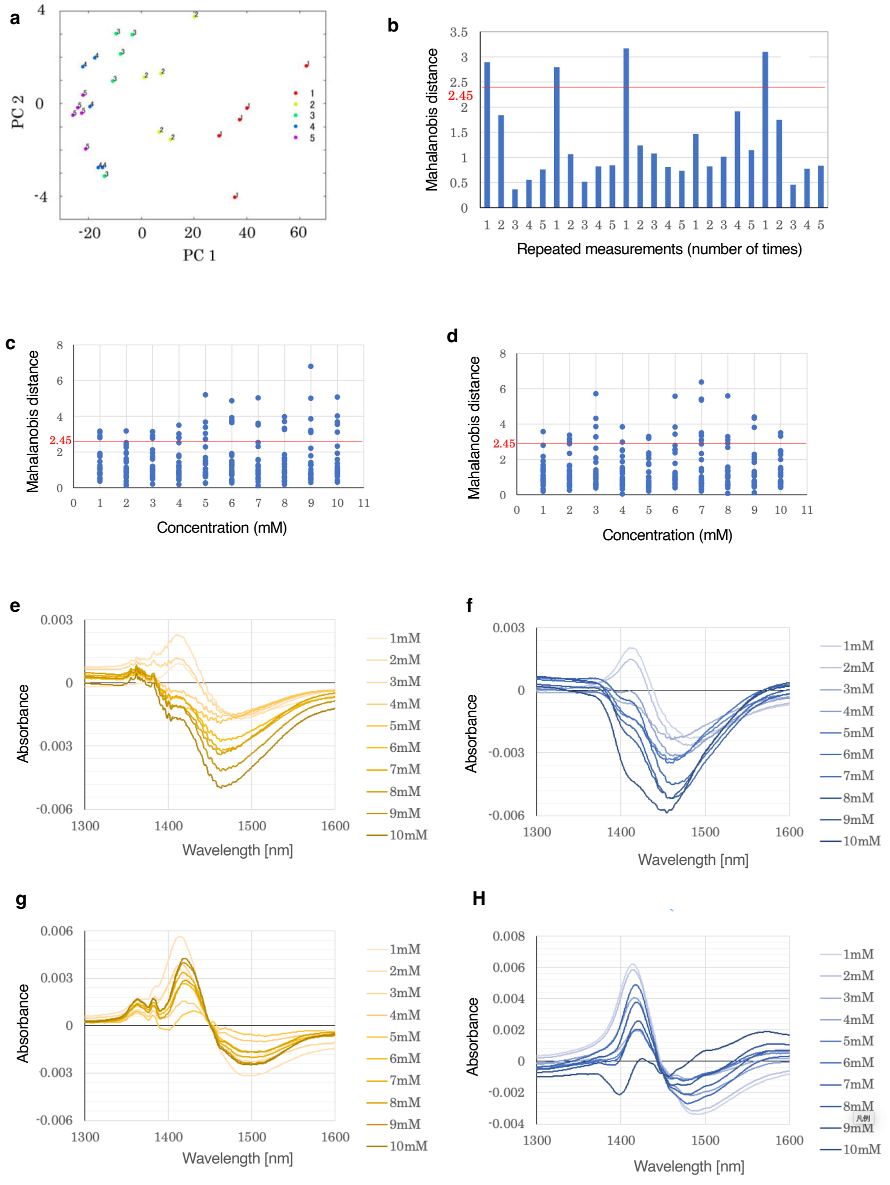

Figure S1: Principal Component Analysis, Mahalanobis Distance Analysis, and Preprocessing of GSH and GSSG Spectral Data (a) Principal component analysis (PCA) results of GSH (1 mM). The x-axis represents the first principal component (PC1), and the y-axis represents the second principal component (PC2). The numbers in the figure indicate the measurement iteration (1st, 2nd, etc.), with five repeated measurements per sample across five independent experiments. (b) Mahalanobis distance analysis of GSH (1 mM). The calculated Mahalanobis distances vary depending on the number of repeated measurements, which is consistent with the PCA results in (a). The threshold for outlier detection was set at 2.45 based on the chi-square distribution, leading to the exclusion of the first measurement as an outlier. (c) Outlier removal using Mahalanobis distance for GSH (1 mM–10 mM). (d) Outlier removal using Mahalanobis distance for GSSG (1 mM–10 mM). (e) Preprocessed spectral data of GSH using only the differential spectral method. (f) Preprocessed spectral data of GSSG using only the subtracted spectra. (g) Preprocessed spectral data of GSH after normalization, smoothing, and subtracted spectra. (h) Preprocessed spectral data of GSSG after normalization, smoothing, and subtracted spectra.

Figure S2

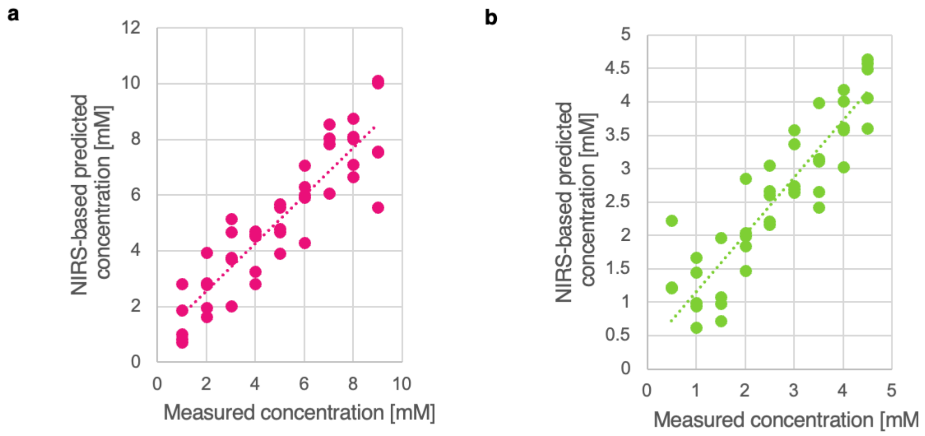

c

	GSH	GSSG
R^2	0.82	0.82
Root means square errors (mM)	0.81	0.40

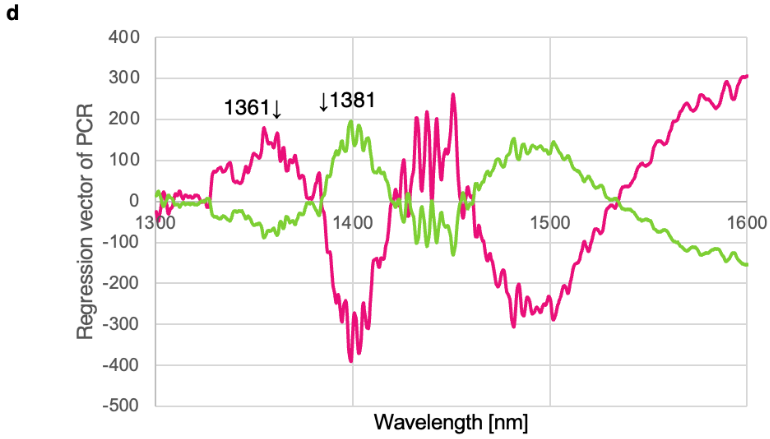

Figure S2: The NIR spectra show differences between GSH and GSSG in the mix solutions through the PCR analysis. (a) Y-fit for prediction of GSH concentration in the mixed sample. (b) Y-fit for prediction of GSSG concentration in the mixed sample. (c) The predictive accuracy of the developed PCR model was high, with determination coefficients of 0.82. Root Mean Square Error (RMSE) values were 0.81 mM for GSH and 0.40 mM for GSSG. (d) Regression vector of GSH (red line) and GSSG (green line) in PCR.

Figure S3

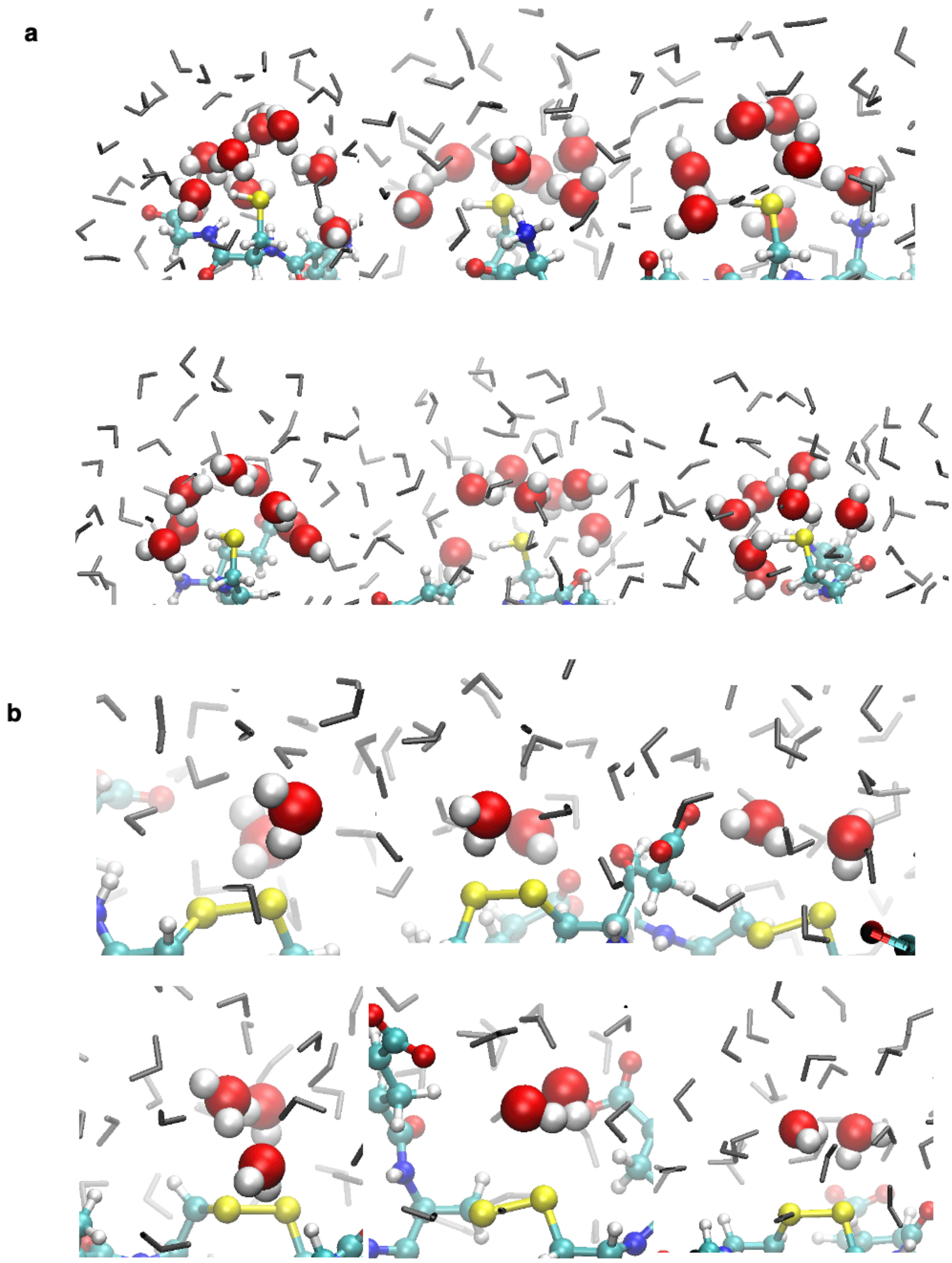

Figure S3: Snapshots of GSH/GSSG models around sulfur atoms (a: GSH, b: GSSG). Yellow, red, blue, cyan, and white circles represent sulfur, oxygen, nitrogen, carbon, and hydrogen atoms, respectively. Water molecules close or far from the sulfur atom are shown in space-filling (red/white) and stick models (gray).
